# Changes in integrin αv, vinculin and connexin43 in the medial prefrontal cortex in rats under single-prolonged stress

**DOI:** 10.3892/mmr.2014.3030

**Published:** 2014-12-02

**Authors:** YANA LI, FANG HAN, YUXIU SHI

**Affiliations:** 1 Department of Histology and Embryology, Institute of Pathology and Pathophysiology, Basic Medical Sciences College, China Medical University, Shenyang, Liaoning 110001, P.R. China; 2 Department of Histology and Embryology, Binzhou Medical University, Yantai, Shandong 264003, P.R. China

**Keywords:** post-traumatic stress disorder, medial prefrontal cortex, integrin αv, vinculin, connexin43, neuronal apoptosis

## Abstract

Post-traumatic stress disorder (PTSD) is a stress-accociated mental disorder that occurs as a result of exposure to a traumatic event, with characteristic symptoms, including intrusive memories, hyperarousal and avoidance. The medial prefrontal cortex (mPFC) is known to be significantly involved in emotional adjustment, particularly introspection, inhibition of the amygdala and emotional memory. Previous structural neuroimaging studies have revealed that the mPFC of PTSD patients was significantly smaller when compared with that of controls and their emotional adjustment function was weakened. However, the mechanisms that cause such atrophy remain to be elucidated. The aim of the present study was to elucidate the possible mechanisms involved in apoptosis induced by single-prolonged stress (SPS) in the mPFC of PTSD rats. SPS is an animal model reflective of PTSD. Of the proposed animal models of PTSD, SPS is one that has been shown to be reliably reproducible in patients with PTSD. Wistar rats were sacrificed at 1, 4, 7 and 14 days after exposure to SPS. Apoptotic cells were assessed using electron microscopy and the TUNEL method. Expression of integrin αv, vinculin and connexin43 were detected using immunohistochemistry, western blotting and reverse transcription polymerase chain reaction. The present results demonstrated that apoptotic cells significantly increased in the mPFC of SPS rats, accompanied with changes in expression of integrin αv, vinculin and connexin43. The present results indicated that SPS-induced apoptosis in the mPFC of PTSD rats and the mitochondrial pathway were involved in the process of SPS-induced apoptosis.

## Introduction

Post-traumatic stress disorder (PTSD) is a mental and behavioral disorder that occurs as a result of exposure to traumatic events. Patients exhibit a number of characteristic symptoms, including avoiding stimuli that they associate with the trauma, continuously re-experiencing the traumatic event, a general numbing of responsiveness and hyperarousal (1,2). Following exposure to the danger of injury or death PTSD may develop and seriously affect the patient’s quality of life and social stability (3). The pathophysiology of PTSD has been widely studied in neuroscience. However, the underlying mechanisms behind PTSD are yet to be elucidated.

Numerous studies have revealed that the amygdala, hippocampus and medial prefrontal cortex (mPFC) are closely associated with the occurrence of PTSD (4). The mPFC is a higher-order structure that controls the stress and fear responses of the amygdala and the hippocampus (5). Computed tomography and functional magnetic resonance imaging have confirmed that the mPFC in the brains of patients with PTSD is significantly smaller than that of healthy individuals, and that their emotional adjustment function is weakened (6,7). A previous study have also confirmed that the aerobic function of the prefrontal cortex in a rat model of PTSD was reduced, the neuronal mitochondria were destroyed, cytochrome oxidase release was enhanced and the neurons were damaged (8). In addition, the study indicated that the mPFC neurons of PTSD rats underwent apoptosis, leading to changes in the structure of the mPFC and a decrease in its volume, which may cause a functional decline. It has been reported that neuronal apoptosis of the amygdala, hippocampus and mPFC are associated with the pathogenesis of PTSD (9,10).

Apoptosis is a genetically programmed, morphologically distinct type of cell death, which is triggered by a range of pathological and physiological stimuli. Integrins are transmembrane proteins that are expressed in neurons and are involved in numerous physiological and pathological processes within the central nervous system (11). A previous study found that in neurodegenerative diseases, integrin αv was closely associated with synaptic function disorders, changes in plasticity, long-term potentiation inhibition and the death and regeneration of neurons (12). Focal adhesion proteins activate the integrin-mediated signal transduction pathway between the extracellular matrix (ECM) and cytoskeletal proteins, thus regulating the physiological functions of a number of cells, including neurons (13). Changes in cytoskeletal proteins affect cell function, and may activate the suicide program of neurons, causing apoptosis. In addition, these changes may affect apoptosis through gap junctional communication among cells. Under this effect, signals initiating apoptosis are diffused through gap junction transfer, thus accelerating the distribution of apoptosis.

Considering the complexity of conducting studies of PTSD in humans, a number of animal models have been developed to replicate the various kinds of trauma that may cause PTSD. Single-prolonged stress (SPS) is a reliable animal model of PTSD based on the time-dependent dysregulation of the hypothalamic-pituitary-adrenal axis, and as such it has been developed and employed for use in PTSD studies (14,15). In effort to elucidate the mechanisms underlying the PTSD-associated reduction in function of the mPFC, the current study employed the rat SPS model to investigate the changes in neuronal apoptosis and the expression levels of integrin αv, vinculin and connexin43 in the mPFC in order to ascertain the correlation between three proteins and neuronal apoptosis. In addition, these observations may provide an experimental basis for further investigation of the mechanism of PTSD.

## Materials and methods

### SPS model and experimental groups

The Wistar rats were supplied by and were conventionally housed in the Experimental Animal Center of China Medical University (Shenyang, China). The rats were housed individually in clear polycarbonate cages (46×24×20 cm) for one week prior to the experiments. All rats were habituated to their cage and given standard food pellets and water. They were housed under a reversed 12 h:12 h light/dark cycle (lights off at 10.00 am), at an ambient temperature (23±2°C) with a humidity of 55±5%. A total of 120 male Wistar rats (7–8 weeks, 150–180 g), were randomly divided into a control and four SPS groups (1, 4, 7 and 14 days post-SPS). The models of PTSD were established using SPS as determined by international PTSD scientific meetings (2). Briefly, the rats were detained for 2 h and immediately underwent a 20-min forced swim in 25°C water in a 40-cm deep tub. Following a 15 min rest period, the rats were anesthetized with ethyl ether (Suzhou Jin Pure Chemical, Co., Ltd., Suzhou, China) until loss of consciousness. All animal experiments were performed in accordance with the guidance suggestions for the Care and Use of Laboratory Animals were supplied by the Ministry of Science and Technology of the People’s Republic of China and approved by the welfare and ethics committee of experimentation, China Medical University. The present study was approved by the ethics committee of the National Natural Science Foundation of China (no. 81171282).

### Transmission electron microscope

The rats of each group were anesthetized with 10 % chloral hydrate (Suzhou Jin Pure Chemical Co., Ltd.)*.* The hearts were exposed, and the left ventricles were perfused with 200–300 ml of 0.9% saline via a catheter through the ascending aorta until a colorless infusion was achieved, followed by perfusion with 300 ml of 4% paraformaldehyde (Suzhou Jin Pure Chemical Co., Ltd.). The whole brains were rapidly removed and dissected on ice, followed by 6–10 h of post-fixation in 4% paraformaldehyde at 4°C. Following being immersed in 20% sucrose solution, glutaraldehyde-fixed (Alfa Aesar China Chemical Co., Ltd., Shanghai, China) mPFC samples were dehydrated with gradient ethanol and acetone (Suzhou Jin Pure Chemical Co., Ltd.) and were embedded with EPON 812 (SPI Supplies, West Chester, PA, USA) epoxy resin. The 70 nm-thick ultrathin sections were stained with uranyl acetate (Shanghai Resonance Biological Science and Technology Co., Ltd., Shanghai China) and lead citrate (Suzhou Jin Pure Chemical Co., Ltd.) and were observed with JEOL1200EX at 100KV.

### Light microscopy

Formaldehyde (Suzhou Jin Pure Chemical Co., Ltd.)-fixed mPFC samples were embedded in paraffin (Shanghai Hua Ling rehabilitation Machinery Factory, Shanghai China) and sliced into 10 μm thick slices for light microscopy.

### Terminal deoxynucleotidyl transferase dUTP nick end labeling (TUNEL) method

The TUNEL staining was performed according to the manufacturer’s instructions (KeyGen Biotech Co., Ltd., Nanjing, China). The apoptosis positive cells were counted under a high-magnification microscope (860; Olympus, Tokyo, Japan).

### Immunohistochemistry for integrin αv, vinculin and connexin43

Immunohistochemical staining was performed using PV two-step immunohistochemical detection kit (Beijing Shan Jinqiao Biological Technology Co., Ltd., Beijing China), integrin αv, vinculin and connexin43 positive cells were detected using mouse anti-rat integrin αv monoclonal antibody (1:50), mouse anti-rat vinculin monoclonal antibody (1:300) and rabbit anti-connexin43 polyclonal antibody (1:200) (Santa Cruz Biotechnology, Inc., Santa Cruz, CA, USA) respectively. Briefly, sections were incubated with 3% H_2_O_2_ at 37°C for 30 min, repaired by microwave, blocked with dripped 10% goat serum for 30 min, incubated with primary antibody at 4°C overnight, visualized with a PV two-step immunohistochemical detection kit, and re-stained with hematoxylin (Suzhou Jin Pure Chemical Co., Ltd.). The Image-Pro Plus image analysis system (Media Cybernetics, Inc., Rockville, MD, USA) was used to analyze the average optical density (OD).

### Western blot analysis for integrin αv, vinculin and connexin43

Briefly, the mPFC homogenates were prepared and the protein concentration was determined using the Coomassie Brilliant Blue method. The homogenates (20 μg) were separated by 10% sodium dodecyl sulfate-polyacrylamide gel electrophoresis (SDS-PAGE) on a condensate gel and the protein was transferred to a PVDF membrane (Millipore, Bedford, MA, USA). Integrin αv (1:300), vinculin (1:1,500) and connexin43 (1:1,200) antibodies were used to probe, respectively. The signals were detected using horseradish peroxidase-labeled immunoglobulin G (1:200) and enhanced chemiluminescence methods. Three bands were subjected to semi-quantitative analysis.

### Semi-quantitative reverse transcription polymerase chain reaction (RT-PCR) detection for integrin αv and connexin43

Total RNA was extracted from the mPFC cells using TRIzol^®^ (Life Technologies, Carlsbad, CA, USA). The purity and concentration of the RNA was detected using the analyzer. Reverse transcription was conducted using the RNA PCR kit (AMV) Ver 3.0 according to the manufacturer’s instructions (Takara Bio, Inc., Otsu, Japan). The specific primers were synthesized by Shenggong Biological Engineering Technology and Services Co., Ltd. (Shanghai, China). The primer sequences used for PCR amplification are presented in Table I. PCR products were semi-quantified on 1% agarose gel using electrophoresis, and the density of each band was analyzed on the Gel Image Analysis system (Tanon 2500R; Tanon, Shanghai, China). The levels of integrin αv and connexin43 mRNA were normalized to β-actin.

### Statistical analysis

The experimental results were analyzed using the SPSS statistical package version 18.0 (SPSS, Inc., Chicago, IL, USA). Data obtained were expressed as the means ± standard deviation. Data analysis among groups was performed using one-way analysis of variance. P<0.05 was considered to indicate a statistically significant difference.

## Results

### Morphological changes observed in neurons using transmission electron microscopy (TEM)

**Figure 1:**
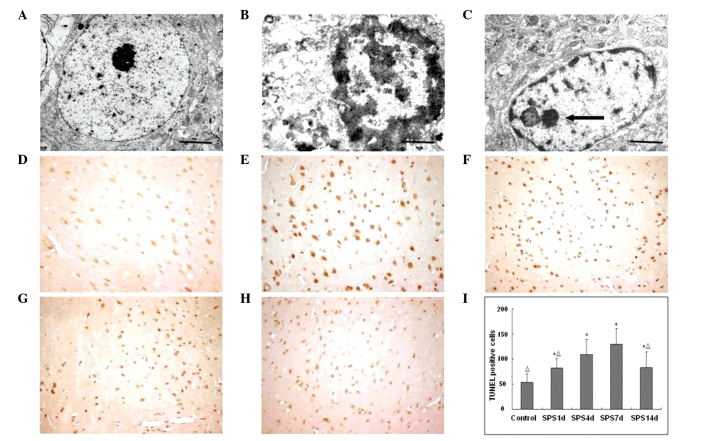
(A–C) Transmission electron microscopy revealed morphological changes in the neurons of the mPFC. (A) Control; (B) SPS 7 day; and (C) SPS 14 day groups; (scale bar, 1 μm). (D–I) Apoptotic cells in the mPFC by TUNEL staining. (D) Control; (E) SPS 1 day; (F) SPS 4 day; (G) SPS 7 day; and (H) SPS 14 day groups (magnification, ×400). (I) Quantification of apoptotic cells. Data are expressed as the mean ± standard deviation. ^*^P<0.05 compared with the control group, ^#^P<0.05 compared with the SPS 7 day group. mPFC, medial prefrontal cortex; SPS, single-prolonged stress; TUNEL, terminal deoxynucleotidyl transferase-mediated dUTP nick end labeling.

Under TEM, normal mPFC neurons had a clear neuronal shape, an intact nuclear membrane and nucleolus, evenly distributed chromatin and normal organelle structure. Following SPS stimulation, the ultrastructures of the neurons of mPFC rats had varying degrees of changes. These included early morphological changes such as cell shrinkage, condensed cytoplasm and shrunken chromatin in the form of a set of edges in the nuclear membrane under the crescent-shaped bodies. With the passage of time, the nucleus degenerated and the nuclear membrane subsided, followed by generation of obvious folds of nuclear cleavage fragments. The majority of the vesicles within the cytoplasm and cell membrane disappeared, and in their place apoptotic bodies appeared (Fig. 1A–C).

### Apoptotic index detected using TUNEL staining

Brown or tan particles present in the nucleus under microscopy were cells that stained positive for apoptosis. The control group showed a small number of apoptotic cells and a lighter color. The number of apoptotic cells in the mPFC of PTSD rats was significantly greater than that of the control group. The apoptotic cells had an irregular shape, uneven size and a darker nucleus color. At ~7 days following SPS stimulation, the number of apoptotic cells reached a peak and then gradually reduced. The number of apoptotic positive cells in each group was compared with that of the control group and the difference was estimated to be statistically significant (Fig. 1).

### Integrin αv, vinculin and connexin43 detection using immunohistochemical staining

**Figure 2:**
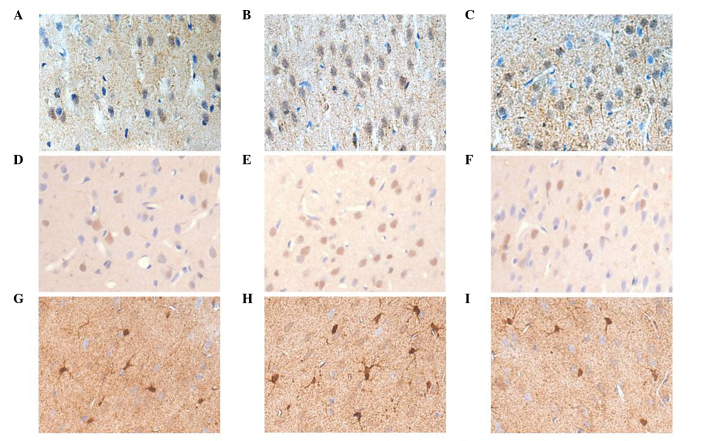
(A–C) Immunohistochemical staining for integrin αv in the mPFC. (A) Control, (B) SPS 4 day, and (C) SPS 7 day groups. (D–F) Immunohistochemical staining for vinculin in mPFC. (D) Control, (E) SPS 4 day, and (F) SPS 7 day groups. (G–I) Immunohistochemical staining for connexin43 in the mPFC. (G) Control, (H) SPS 4 day, and (I) SPS 7 day groups. mPFC, medial prefrontal cortex; SPS, single-prolonged stress.

**Figure 3:**
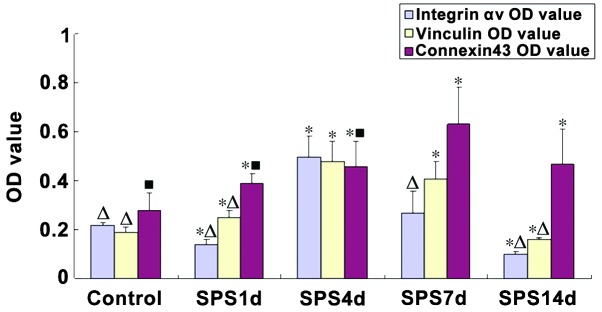
Quantitative analysis of the average ODs of integrin αv, vinculin and connexin43 as determined by immunohistochemistry in the medial prefrontal cortex. Data are represented as the mean ± standard deviation. ^*^P<0.05 compared with control, ^Δ^P<0.05 compared with SPS 4 day, ^=^P<0.05 compared composed with SPS 7 day. SPS, single-prolonged stress; OD, optical density.

Integrin αv and vinculin demonstrated a weak positive reaction in normal rat mPFC neurons with a relatively light staining, which were primarily distributed in the cytomembrane and the cytoplasm of mPFC neurons. Following SPS stimulation, the expression levels of integrin αv markedly decreased on day 1, following which it began to increase, reaching a peak on day 7. In the SPS rats, increased vinculin levels were observed compared with those of the controls, with the highest expression levels were identified 4 day after SPS stimulation. However, the expression levels of integrin αv and vinculin markedly reduced on day 11 in the SPS group rats. Positive immunohistochemical cells stained with the antibody against connexin43 were brown. The immunoreactivity against connexin43 was primarily observed in the cytoplasm and neurite of astrocytes. There were statistically significant differences in the connexin43 expression levels between the control and SPS groups (P<0.05). In the SPS groups, the expression of connexin43 gradually increased from day 1, reaching a peak on day 7 (Figs. 2 and 3).

### Western blot analysis of integrin αv, vinculin and connexin43

**Figure 4:**
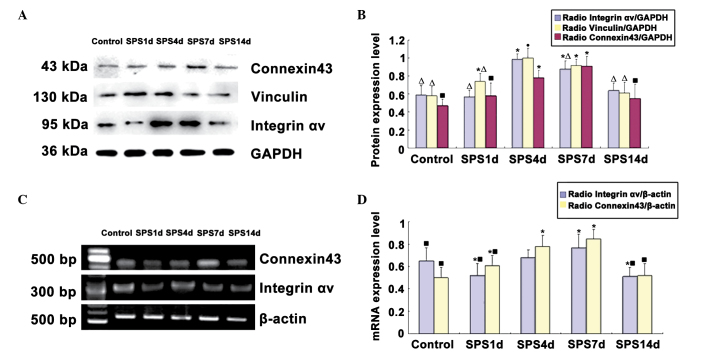
Western blot analysis and RT-PCR analysis for integrin αv, vinculin and connexin43 in the medial prefrontal cortex. (A) Western blot analysis. (B) Quantification of the western blot analysis. (C) RT-PCR analysis. (D) Quantification of RT-PCR analysis. Data are presented as the mean ± standard deviation. ^*^P<0.05 compared with the control group, ^Δ^P<0.05 compared with the SPS 4 day group, ^=^P<0.05 compared with the SPS 7 day group. RT-PCR, reverse transcription polymerase chain reaction; SPS, single-prolonged stress.

The integrin αv, vinculin, connexin43 and glyceraldehyde 3-phosphate dehydrogenase (GAPDH) immunoreactive signals appeared at 95, 130, 43 and 36 kDa, respectively, and the mean values of the band densities of the control group were set as 100%. Data were expressed with normalized OD. Following SPS stimulation, the protein levels of integrin αv were downregulated at 1 day and then recovered gradually compared with those of the control group. Analysis of the expression levels of vinculin and connexin43 protein revealed a significant increase in the SPS groups compared with the levels in the control group (P<0.05). The intensity of vinculin reached a peak on day 4. The expression of connexin43 protein reached its highest level on day 7. The time course of the western blot analysis results was consistent with the findings obtained by immunohistochemical analysis (Fig. 4).

### mRNA expression of integrin αv and connexin43

The mRNA levels of integrin αv and connexin43 were normalized to the β-actin mRNA level. The integrin αv mRNA expression levels in the mPFC were significantly reduced on day 1 of the SPS group compared with the control group (P<0.05). The levels increased gradually and peaked on SPS day 7. The integrin αv mRNA expression changed over time, which was consistent with the results of immunohistochemistry and western blot analysis. However, the mRNA expression levels of connexin43 markedly increased in the SPS group rats compared with the control group, reaching a peak on day 7 (Fig. 4).

## Discussion

PTSD is a psychiatric disorder, which may occur as a result of experiencing or witnessing life-threatening events, including military combat, natural disasters, terrorist incidents or violent personal assaults (16). A study has shown that patients with PTSD exhibit a dietary status change, enhanced negative feedback inhibition, enhancement of the acoustic startle response and impaired spatial memory (17). Further studies have reported that patients with PTSD have a smaller amygdala and hippocampus (18,19). In addition, our previous studies have confirmed that the amygdala and hippocampal neurons of PTSD rats displayed signs of apoptosis (20,21). The varying degrees of PTSD mental symptoms may be caused by inadequate descending inhibition of reduced mPFC function to the amygdala (22).

In the current study, transmission electron microscopy revealed that the mPFC neurons in PTSD model rats underwent apoptosis. In addition to the extension of SPS stimulation time, the number of decrescent neuronal cell bodies increased. Through TUNEL staining, the apoptotic index was found to be significantly increased following SPS stimulation compared with that of the controls. In addition, TUNEL indicated that the mPFC neurons of PTSD rats underwent apoptosis, leading to changes in the structure of the mPFC and a reduction in its volume causing functional decline. This exhibits a certain association with the pathogenesis of PTSD.

Apoptosis, also known as programmed cell death, is a programmed cell death process that is regulated by genes (23,24). The results of the current study have confirmed that factors that trigger apoptosis transfer certain stimulus signals to cells by the way of transmembrane information transfer to connect the entire death program, causing apoptosis (25). The integrin family is a class of transmembrane heterodimeric glycoprotein adhesion molecules. They are involved in cell signaling, and regulating cell survival and apoptosis (26). Integrin αv is an important member of the integrin family. It can regulate neuronal function and mediate astrocyte adhesion and migration. A previous study found that the subunit ectodomain of integrin αv could identify arginine-glycine-aspartic acid sequences of the ECM, while the intracellular domain integrated intracellular and extracellular signals via the interaction with cytoskeletal cross-linker proteins, including the signaling pathway-related proteins vinculin, α-actin and talin, which have an important role in the regulation of cell adhesion, proliferation, differentiation, invasion, migration and apoptosis (27,28). Vinculin is a cytoskeletal protein and a focal adhesion protein, mainly located in cell-cell junctions and cell-ECM focal adhesion sites (29,30). Vinculin is joined to the microfilament cytoskeleton of cells, anchoring the microfilament to the cell membrane, and having an important role in the maintenance of cell morphology and the regulation of cell adhesion, motility, proliferation and survival (31). In addition, vinculin, as an important component of focal adhesions, is involved in the cytomechanics and chemistry of the integrin-mediated signal transduction. Integrins can promote vinculin activation when in contact with the ECM. Vinculins are raised to focal adhesions where the integrins are located, causing the polymerization and reorganization of cytoskeleton actins, therefore they have an important role in cell connection and fixation (20). Connexin43 is primarily expressed in astrocytes in the brain. It can connect a large number of astrocytes together, allowing for the mutual circulation of various ions, small molecular substances and metabolites, forming functional coupling syncytia (32). Therefore, it can rapidly dilute extracellularly transported neurotransmitters and metabolites, remove metabolites gathered around neurons in time, weakening the damage caused by metabolites to the glial cells themselves, and having an important role in maintaining the stability of the brain tissue microenvironment and the coordination of neuronal functions (33).

In the current study, the location and expression of integrin αv, vinculin and connexin43 in the mPFC of PTSD rats were observed using immunohistochemical staining, and semi-quantitative analysis was conducted using western blot analysis and RT-PCR. The results revealed that integrin αv expression is significantly decreased following SPS stimulation. The reduction in the integrin αv expression levels may lead to the loss of the connection between astrocytes and the ECM, or between astrocytes, damages of focal adhesion structures, changes in the structures of cytoskeleton proteins, breakdown of the signal transduction pathways of cell growth and the proliferation activation of apoptosis signal transduction, and eventually astrocyte shrinkage, isolation, and apoptosis (34). Expression levels of vinculin and connexin43 in the mPFC of PTSD rats demonstrated a transient increase. This may be due to the fact that following stress stimulation, the increased cellular stress and enhanced adhesion increase cell resistance to injury, therefore the number of astrocytes increases, triggering more abundant intercellular gap junctions, resulting in a timely removal or buffer of the stress factors, cytokines and excitatory neurotransmitters produced following stress, thereby taking a compensatory protective effect on neurons. In the time period following the SPS stimulation, the structure of neurons and glial cells was further damaged, cell adhesion to the ECM was reduced, the number of gap junctions between the cells gradually reduced and the gene and protein expression levels of vinculin and connexin43 were significantly reduced. The reduction in the expression level of vinculin may reduce the mechanical strength of the integrin-cytoskeleton connection, increase cell motility and reduce focal adhesions, leading to reduced cell adhesion. Meanwhile, changes in the cytoskeleton and the reduction and depolymerization of microfilaments and microtubules promote the reduction of the force of cells binding with ECM, leading to apoptosis (34,35). The reduction in connexin43 expression results in brain tissue microenvironment and cell communication disorders, ultimately leading to mPFC dysfunction of PTSD rats.

In conclusion, the pathogenesis of PTSD has not yet been completely elucidated. The stressor causes structural changes in the mPFC of the brain and neuronal apoptosis, leading to the onset of PTSD. However, the detailed pathogenesis requires further study.

## Figures and Tables

**Table I:** Primers for integrin αv, connexin43 and β-actin.

Gene	Primer	Product size (bp)
Integrin αv	Sense: 5′-TCGTTTCTATCCCACCGC-3′ Antisense: 5′-GGCTTTCCTTGTGCTCCC-3′	366
Connexin43	Sense: 5′-AAAGGCGTTAAGGATCGCGTG-3′ Antisense: 5′-GTCATCAGGCCGAGGCCT-3′	438
β-actin	Sense: 5′-GTCACCCACACTGTGCCCATCT-3′ Antisense: 5′-ACAGAGTACTTGCGCTCAGGAG-3′	542
